# Telomere Architecture Correlates with Aggressiveness in Multiple Myeloma

**DOI:** 10.3390/cancers13081969

**Published:** 2021-04-19

**Authors:** Aline Rangel-Pozzo, Pak Lok Ivan Yu, Sadhana LaL, Yasmin Asbaghi, Luiza Sisdelli, Pille Tammur, Anu Tamm, Mari Punab, Ludger Klewes, Sherif Louis, Hans Knecht, Adebayo Olujohungbe, Sabine Mai

**Affiliations:** 1 Cell Biology, Research Institute of Oncology and Hematology, University of Manitoba, CancerCare Manitoba, Winnipeg, MB R3C 2B1, Canada; ivan.p.l.yu@alumni.ubc.ca (P.L.I.Y.); Sadhana.lal@gmail.com (S.L.); asbaghiy@myumanitoba.ca (Y.A.); l.sisdelli@unifesp.br (L.S.); lr_klewes@yahoo.ca (L.K.); 2 Department of Medicine, University of British Columbia, Vancouver, BC V6T 1Z4, Canada; 3 Genetic Bases of Thyroid Tumors Laboratory, Division of Genetics, Department of Morphology and Genetics, Universidade Federal de São Paulo, São Paulo 04082-001, Brazil; 4 Department of Clinical Genetics, United Laboratories, Tartu University Hospital, 50406 Tartu, Estonia; Pille.Tammur@kliinikum.ee; 5 United Laboratories, Tartu University Hospital, 50406 Tartu, Estonia; anu.Tamm@kliinikum.ee; 6 Department of Haematology and Bone Marrow Transplantation, Clinic of Haematology and Oncology, Tartu University Hospital, 50406 Tartu, Estonia; Mari.Punab@kliinikum.ee; 7 Telo Genomics Corp, Toronto, ON M5G1L7, Canada; sherif.louis@telodx.com; 8 Division of Hematology, Department of Medicine, Jewish General Hospital, McGill University, Montréal, QC H3T 1E2, Canada; Hans.Knecht@mcgill.ca; 9 Haematology, CancerCare Manitoba, Winnipeg, MB R3C 2B1, Canada

**Keywords:** multiple myeloma, monoclonal gammopathy of undetermined significance, telomeres, survival, structural genome markers, genomic instability, 3D imaging

## Abstract

**Simple Summary:**

Multiple myeloma (MM) remains an incurable blood cancer. One of the current challenges in patient management is the risk assessment and subsequent treatment management for each patient with MM. Patients with an identical diagnosis may present very different disease courses and outcomes. This challenge of MM is a current focus of the scientific and medical communities. In our research, we have used an imaging approach to determine the risk of MM patients to progressive/aggressive disease. Using three-dimensional (3D) imaging of telomeres, the ends of chromosomes, we report that specific telomeric profiles are associated with aggressive disease.

**Abstract:**

The prognosis of multiple myeloma (MM), an incurable B-cell malignancy, has significantly improved through the introduction of novel therapeutic modalities. Myeloma prognosis is essentially determined by cytogenetics, both at diagnosis and at disease progression. However, for a large cohort of patients, cytogenetic analysis is not always available. In addition, myeloma patients with favorable cytogenetics can display an aggressive clinical course. Therefore, it is necessary to develop additional prognostic and predictive markers for this disease to allow for patient risk stratification and personalized clinical decision-making. Genomic instability is a prominent characteristic in MM, and we have previously shown that the three-dimensional (3D) nuclear organization of telomeres is a marker of both genomic instability and genetic heterogeneity in myeloma. In this study, we compared in a longitudinal prospective study blindly the 3D telomeric profiles from bone marrow samples of 214 initially treatment-naïve patients with either monoclonal gammopathy of undetermined significance (MGUS), smoldering multiple myeloma (SMM), or MM, with a minimum follow-up of 5 years. Here, we report distinctive 3D telomeric profiles correlating with disease aggressiveness and patient response to treatment in MM patients, and also distinctive 3D telomeric profiles for disease progression in smoldering multiple myeloma patients. In particular, lower average intensity (telomere length, below 13,500 arbitrary units) and increased number of telomere aggregates are associated with shorter survival and could be used as a prognostic factor to identify high-risk SMM and MM patients.

## 1. Introduction

Multiple myeloma (MM) is a B-cell malignancy characterized by the extensive proliferation of malignant plasma cells (PCs) in the bone marrow (BM) and an abnormal increase in monoclonal immunoglobulins or M proteins [1]. This aberrant plasma cell proliferation leads to lytic bone lesions, hypercalcemia, kidney failure, and severe anemia [1]. MM is the end stage of monoclonal gammopathy of undetermined significance (MGUS) and smoldering multiple myeloma (SMM) [2,3]. All cases of MM are preceded by MGUS or SMM [2,3]. MGUS patients have detectable monoclonal immunoglobulin in blood or urine (<3 g/dL), but no evidence of end-organ damage or other symptoms. The risk of progression from MGUS to MM is approximately 1–2% per year [4,5]. In SMM patients, the levels of M protein in the serum are higher than in MGUS (≥3 g/dL), with patients still showing no symptoms and no laboratory signs of end-organ damage, but the risk of progression to MM is approximately 10% per year in the first 5 years [4,5]. High-risk SMM patients are estimated to progress to active myeloma within 2 years of diagnosis and have genetic features that promote faster progression to the active stage of the myeloma disease [6,7].

Despite the development of novel therapeutic strategies and a better understanding of MM biology, this disease remains incurable with a heterogeneous clinical course, where overall survival can range from a few months to over 10 years [8]. Therefore, the development of early reliable surrogate end-points for survival is necessary for better risk stratification, treatment individualization, and the future incorporation of novel agents into MM management. Among the many prognostic factors and several different risk stratification systems described for MM, the International Staging System (ISS), initially based on serum levels of both β2-micoglobulin and albumin [9], incorporated cytogenetic abnormalities and lactate dehydrogenase levels in 2005 [9]. However, gene expression profile, plasma cell proliferative rate, extramedullary disease, initial presentation as plasma cell leukemia, age, performance status, PET-CT presentation, and comorbidities are also taken into consideration in the clinical perspective on treatment goals and management of MM patients [9].

A prominent feature observed in MM cells is a dynamic genomic instability and complexity, which increases with the subsequent acquisition of additional genetic abnormalities [6]. The cell-to-cell heterogeneity and the presence of multiple subclones affect both prognostic stratification and therapeutic approaches [10]. This intra-clonal diversity, where different clones are present at diagnosis and during disease evolution, promotes a survival advantage of individual clones upon treatment, selection of minor pre-existing or novel clones, and disease progression [10,11,12,13].

Telomeres have (TTAGGG)n repeat sequences that are capped by “shelterin” proteins [14]. Short dysfunctional telomeres are characterized by insufficient repeats, in which only a small number of shelterin proteins can bind and protect the telomeres [14,15]. Irrespective of telomere length, the impaired/non-existent shelterin binding leads to “uncapped” telomeres that activate the DNA damage response, chromosomal fusion, and anaphase-bridging, leading in turn to breakage–bridge–fusion cycles and dynamic genomic instability with clonal evolution [16,17]. Telomere dysfunction is one of the mechanisms that may lead to the genetic and clinical heterogeneity observed in MM [16,18,19] and could therefore be of prognostic significance. Here, we compared different 3D telomere parameters from bone marrow samples across MGUS, SMM, and MM patients, prior to treatment, and investigated a potential prognostic role of telomere assessment in the spectrum of myeloma at diagnosis.

## 2. Materials and Methods

### 2.1. Sample Acquisition and Patient Population

This study recruited 214 patients from both CancerCare Manitoba and Tartu University Hospital between 2010 and 2014. The study population consisted of treatment-naïve patients with MGUS (*n* = 54), SMM (*n* = 24), and MM (*n* = 136). Bone marrow samples were collected at diagnosis. The researchers were blinded to the disease stage of the samples (MGUS, SMM, or MM) which were analyzed in this study. Available patient clinical characteristics are presented in Supplementary Table S1 (Table S1). This study was approved by the Research Ethics Review Board on Human Studies of the University of Manitoba (Ethics Reference No. H2010:170) and the Ethics Review Committee on Human Research of the University of Tartu (Protocol No. 194T-11) in accordance with the declaration of Helsinki.

### 2.2. Sample Preparation

Bone marrow samples were processed as previously described [18]. The white blood cells were isolated using Ficoll-Paque (GE Healthcare Life Sciences, Baie d’Urfe, QC, Canada). The cells were washed with Roswell Park Memorial Institute (RPMI) medium (Gibco Life Technologies Inc., Burlington, ON, Canada) containing 10% fetal bovine serum (FBS) (Gibco Life Technologies Inc.). The cells were placed onto poly-*L*-lysine coated slides. The cells were then fixed in a 3.7% formaldehyde solution for 20 min and the slides were washed twice with 1× phosphate buffered saline (PBS).

### 2.3. Immunostaining and Telomere Hybridization

The co-immunostaining and 3D telomeres FISH protocol was conducted as previously described [19]. In brief, the cells were blocked with 4% BSA in 4x saline sodium citrate (SSC) and incubated with Alexa Fluor^®^ 488 labelled Mouse Anti-Human CD56 antibody (BD Bioscience, San Jose, CA, USA) and Alexa Fluor^®^ 594 labelled anti-human CD138 (Syndecan-1) (Biolegend, San Diego, CA, USA). Next, for telomere hybridization, the cyanine 3 (Cy3)–labelled peptide nucleic acid probe (DAKO, Glostrup, Denmark) was used [19]. Next, for probe hybridization, we used the HYBrite Denaturation and Hybridization System (Vysis; Abbott Diagnostics, Des Plains, IL, USA). Unbound probe was removed by washing with 70% formamide (Sigma-Aldrich, St Louis, MO, USA)/10 mM Tris (pH 7.4) for thirty minutes, in 0.1× saline sodium citrate (SSC) at 55 °C for five minutes, and twice in 2× SSC/0.05% Tween-20 for five minutes. The nuclei were counterstained with 4′,6-diamidino-2-phenylindole (DAPI) (Sigma-Aldrich, St. Louis, MO, USA) and mounted with VECTASHIELD (Vector Laboratories, Burlington, ON, Canada).

### 2.4. Identification of Malignant Plasma Cells

The malignant plasma cells were differentiated from normal lymphocytes based on their positive double staining for the MM markers CD138 and CD56, augmented nuclear size, and weaker DAPI counterstain, as it has been shown that malignant plasma cells show larger and weaker DAPI-stained nuclei than normal lymphocytes [18,19].

### 2.5. Image Acquisition and Nuclear Architecture Analysis

Fifty CD138^+^/CD56^+^ interphase nuclei were analyzed per sample. The telomeres were imaged using fluorescence microscopy (Zeiss AxioImager Z1 microscope (Carl Zeiss, Toronto, ON, Canada) equipped with an AxioCam HRm camera, using a 63 ×/1.4 oil plan apochromatic objective lens). The imaging software ZEN 2.3 software was used for image acquisition. Three-dimensional imaging of telomeres was performed by acquiring 80 stacks along the *z*-axis (specimen depth), with a 0.2 μm thickness. The exposure time for Cy3 (telomeres) was maintained at 100 milliseconds. The images were deconvolved using a constrained iterative algorithm [20]. After deconvolution, the images were analyzed using TeloView^®^ v1.03 software (Telo Genomics Corp., Toronto, ON, Canada). TeloView^®^ software was used with the permission of Telo Genomics Corp. (Toronto, ON, Canada). TeloView^®^ determined the following 6 telomere parameters: telomere signal intensity (total and average), number of telomere signals, number of telomere aggregates (i.e., clusters of telomeres too close to be further resolved at an optical resolution limit of 200 nm), nuclear volume, a/c ratio (i.e., spatial distribution of the telomeres within the nucleus in a cell cycle-dependent manner), and distribution of telomeres relative to the nuclear periphery [21].

### 2.6. Statistical Analyses

For statistical analysis, the software package SAS × version 9.4 (SAS Institute Inc., Cary, NC, USA) was employed to perform nested factorial analysis of variance in the telomere parameters measured using TeloView^®^. Chi-square tests were used to compare the percentage of interphase telomere signals at each given intensity level at intervals of 1000 intensity units, ultimately divided into quartiles for analysis. Nested factorial analysis of variance was also used to compare the distribution of signal intensities across MGUS, SMM, and MM. Hierarchical centroid cluster analysis was used to identify different clusters of patients. The cluster groups were defined based on combinations of 3D telomere parameters. Univariate comparisons for overall survival (OS) were conducted with the log-rank test and displayed as Kaplan–Meier curves. Cox proportional hazards models were used to estimate hazard ratios (HR) for OS with adjustment for age and diagnosis. A *p*-value of < 0.05 was considered significant.

## 3. Results

### 3.1. Telomere-Related Genomic Instability Differentiates Between MGUS, SMM, and MM and Identifies SMM and MM Patients with Progressive Disease

Basic clinical data for the patients are summarized in Table 1. The average age of the study population was 66.6 years for MGUS, 70.8 years for SMM, and 67.5 years for MM (Table 1). The average percentage of plasma cell bone marrow infiltration was 2.8, 18.1, and 37.9% for MGUS, SMM, and MM, respectively, and the average amount of serum myeloma protein (M-protein) was 5.5 g/L for MGUS, 17.1 g/L for SMM, and 26.9 g/L for MM. In all groups, the majority of patients had the immunoglobulin G isotype (IgG). A small proportion of all groups had the IgA isotype, followed by the IgM subtype. MM was the only group that displayed lytic lesions, as well as a greatly elevated level of serum M-protein compared to MGUS and SMM patients [5]. The most common immunoglobulin isotype in MM patients was IgG, followed by IgA, as previously described [18].

Based on cytogenetic FISH analyses, only two MGUS patients displayed the chromosomal aberrations commonly associated with MM. However, cytogenetic data for t(11;14) were only available for 36 patients of the MGUS group and for 25 patients of the MM group. In addition, t(4;14) and del(14q1.3)/13qter results were only available for eight patients of the MGUS group and for 19 of the MM group (Table S1). Interestingly, three MM patients had both t(11;14) and t(4;14) translocations and three other patients had additionally a 14q13 deletion.

**Figure 1:**
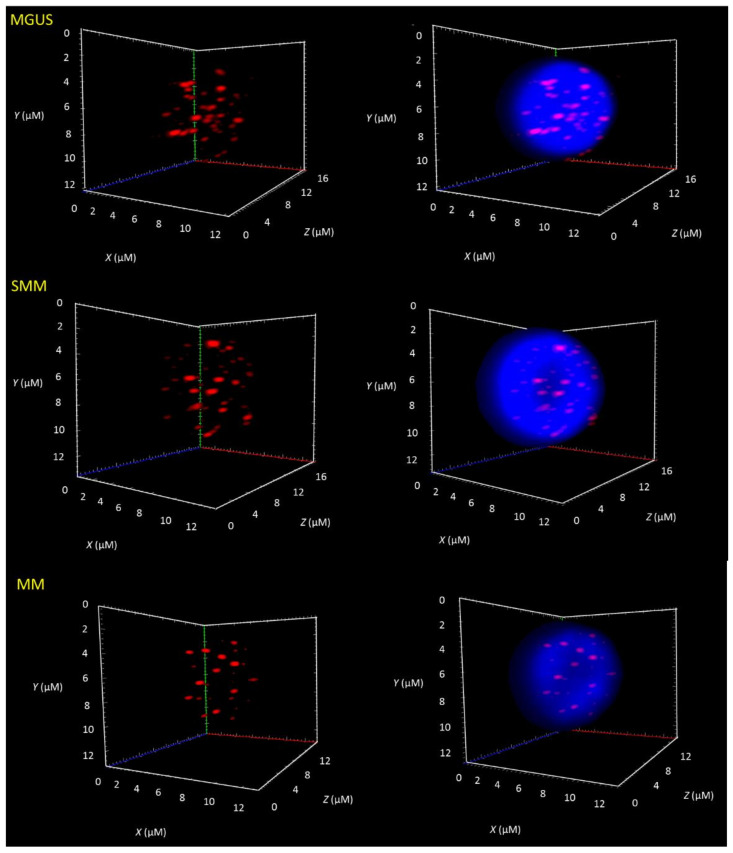
Differences in the 3D nuclear telomere architecture between MGUS, SMM, and MM. Representative nuclei, counterstained with DAPI (blue) from MGUS, SMM, and MM patient samples. The Cy-3 labelled telomeres appear as red signals. Numerous telomere parameters showed alteration along the three disease stages. Most notably, the decrease in telomere number between MM and MGUS is associated with an increase in genomic instability. For additional significant changes, please see Table 2.

Three-dimensional telomere profiles were measured using TeloView^®^ [21], which was used to assess the levels of telomere-related genomic instability and specific 3D telomere parameters associated with disease stability vs. progression [18,22,23,24,25,26]. To assess the potential of 3D telomere architecture as a reliable tool to differentiate MM from its precursor stages, we evaluated a total of 214 patients (54 MGUS, 24 SMM, and 136 MM). For all patients, treatment-naïve bone marrow samples at diagnosis were analyzed. We selected the cells based on their dual positive staining for CD56 and CD138, by their weaker DAPI counterstain, and larger nuclear size when compared to normal lymphocytes (Supplementary Figure S1). The telomeres were visualized as red signals (Figure 1). In Figure 1, we show representative 3D images of CD56^+^/CD138^+^ cells from patients in each disease stage.

Table 2 summarizes the 3D telomere parameters measured by TeloView in MGUS, SMM, and MM as well as in the respective sub-groups of SMM and MM, which include indolent and high-risk SMM and stable vs. progressive MM, respectively. The following 3D telomere parameters are shown in Table 2; changes in telomere numbers, which are indicative of gains or losses of chromosomes and intrachromosomal rearrangements, telomere intensity, which is proportional to telomere length, telomere aggregates, which indicate telomere clusters and/or fusions, and the a/c ratio, which measures cell proliferation. Finally, Table 2 also includes the nuclear volume and telomeres per nuclear volume.

Briefly, telomere numbers showed significant differences between MGUS and MM, between indolent and high-risk SMM, and between stable and progressive MM. No significant differences were noted between MGUS and all SMM without risk grouping. Total telomere intensity was found to significantly differ between MGUS and SMM, in the combined group of SMM, and in the subgroup of indolent/high-risk SMM. The average telomere intensity followed this trend and also included significant differences between stable and progressive MM. Telomeric aggregates became a significant discriminator between indolent and high-risk SMM, as well as between stable and progressive MM. The a/c ratio indicated significant differences in proliferative capacity between MGUS and MM, as well as between stable and high-risk SMM and between stable and progressive MM. Nuclear volumes differed between MGUS and SMM, indolent and high-risk SMM, and stable vs. progressive MM. Telomeres per nuclear volume showed no significance across the myeloma spectrum. In conclusion, telomere numbers, intensity, aggregates, and a/c ratio offer distinctive features throughout the MM spectrum, with significant values as discussed above and shown in Table 2. Thus, the level of genomic instability as measured by the 3D spatial organization of telomeres varies across the MM spectrum, with the highest combined telomere changes found in the SMM and MM subgroups (Table 2).

### 3.2. Stratifying SMM Patients and MM Patients Highlights High-Risk and Progression Groups

Myeloma and its precursor lesions are heterogeneous stages of the diseases. Therefore, stratifying patients with SMM and MM into respective risk groups based on their 5-year clinical follow-up data (progression and survival) is of critical clinical importance (Supplementary Table S2). Regarding the SMM group, patients without follow-up data were excluded, limiting the analysis to a total of 20 SMM patients. Fifteen of the 20 SMM patients remained stable for 5 years, while five progressed to full stage multiple myeloma within 1 to 3 years from the point of diagnosis. They were stratified as indolent SMM (stable SMM) and high-risk SMM (SMM progression), respectively. The disease progression of high-risk SMM patients was confirmed clinically by MM-caused morbidity. The comparison of the 3D telomere profiles between SMM-stable and SMM-progression showed that these were two distinct groups with different levels of telomere-related genomic instability. Five telomere parameters were significantly different, including telomere numbers, intensity (total and average), telomeric aggregates, a/c ratio, and nuclear volume (Table 2).

For the MM group (133 patients with 5 years follow-up), 85 MM patients were with stable disease and 48 MM patients with progressive disease. Total number of signals, average intensity, telomere aggregates, nuclear volume, and a/c ratio showed significant differences: MM patients with progressive disease had increased (↑) telomere signals, telomere aggregates, a/c ratio, and nuclear volume with a decrease in the average intensity of telomere signals in comparison with MM patients with stable disease (Table 2). Basic clinical data for the SMM and MM subgroups are summarized in Table S2.

### 3.3. Telomere Intensity Was Significantly Associated with Shorter Overall Survival in MM

**Figure 2:**
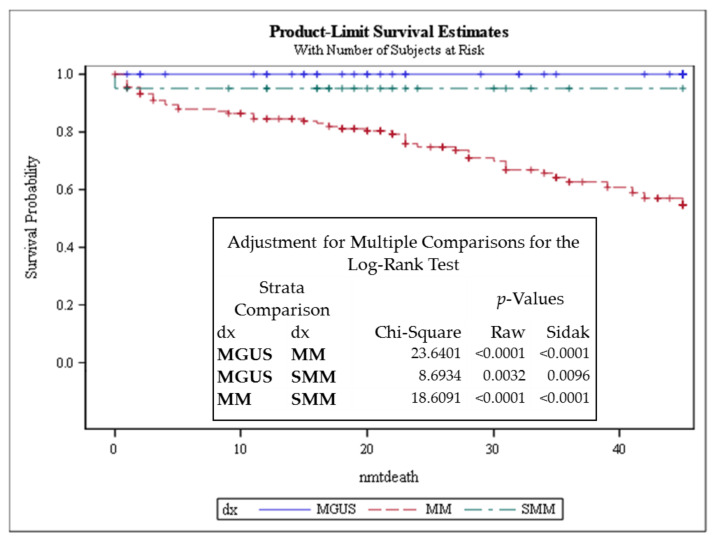
Kaplan–Meier survival analysis performed in patients with MGUS, SMM, and MM using the log-rank test, agreeing with previous reports, where MM patients showed significantly inferior survival compared to SMM and MGUS patients. dx—diagnosis; Strata—different diagnosis; raw *p*-values—*p*-values without adjustments; Sidak *p*-value—*p*-value for multiple comparisons; nmtdeath refers to months to dying censored. Censored refers to cases that were followed up to that point but did not die.

**Figure 3:**
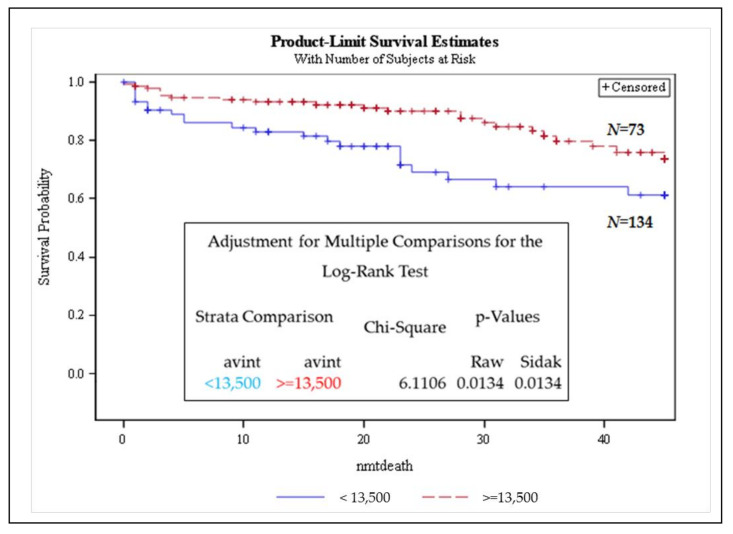
Kaplan–Meier survival analysis performed in patient (MGUS, SMM, and MM) cohorts using the log-rank test. The telomere average intensity threshold (<13,500 a.u) identified a subset of MM patients with inferior survival. dx—diagnosis; Strata—groups; raw *p*-values—*p*-values without adjustments; Sidak *p*-value—*p*-value for multiple comparisons; nmtdeath refers to months to dying censored. Censored refers to cases that were followed up to that point but did not die.

We next investigated whether telomere parameters were of prognostic value for overall survival by performing Cox’s proportional hazards modelling in our cohort (Supplementary Table S2). Kaplan–Meier survival analysis (performed using the log-rank test) showed agreement with previous reports, where MM patient survival was significantly inferior to SMM and MGUS patients (Figure 2). When considering a telomere average intensity threshold (<13,500 fluorescent arbitrary units (a.u)), a subset of MM patients with inferior survival was identified (Figure 3). Average intensity was highly prognostic in multiple myeloma (MM). Patients with a telomere average intensity below 13,500 a.u. had significantly shorter OS when compared to patients above 13,500 a.u. The proportional hazard modelling showed that total intensity and average intensity were significant parameters to predict OS in MM. In multivariate analysis, every predictor was adjusted for the other predictors in the model (Table 3).

The measure of effect is the hazard ratio, which is the risk of failure (i.e., the risk or probability of suffering the event in question). If the hazard ratio is less than 1, then the predictor is protective (i.e., associated with improved survival). On the other hand, if the hazard ratio is greater than 1, then the predictor is associated with increased risk (or decreased survival). The *p* value shows statistically significant associations between the first column parameters with mortality. DF—degree of freedom, each predictor occupies 1 degree of freedom in the model. Avint—average intensity of telomere signals; totin—total intensity of telomere signals; mmdx—MM diagnosis; and smmdx—SMM diagnosis. It is important to note that effects were adjusted for all predictors in the model. For total intensity, the HR was 1.0005 (95% confidence interval (CI), 1.000–1.009), *p* = 0.0297, and for average intensity, the HR was 0.594 (95% confidence interval (CI), 0.467–0.754), *p* < 0.0001. Then, after adjustment for age and diagnosis, both total intensity (totin) and average intensity (avint), summarized in Table 2, continued to be associated with significantly shorter survival. Adjustment for age alone had total intensity HR 1.005, 95% CI 1.000–1.009, *p* = 0.04, and average intensity HR 0.602, 95% CI 0.463–0.784, *p* = 0.0002; while adjustments for age and diagnosis had total intensity HR 1.044, 95% CI 1.000–1.008, *p* = 0.04, and average intensity HR 0.635, 95% CI 0.488–0.827, *p* = 0.0007.

**Figure 4:**
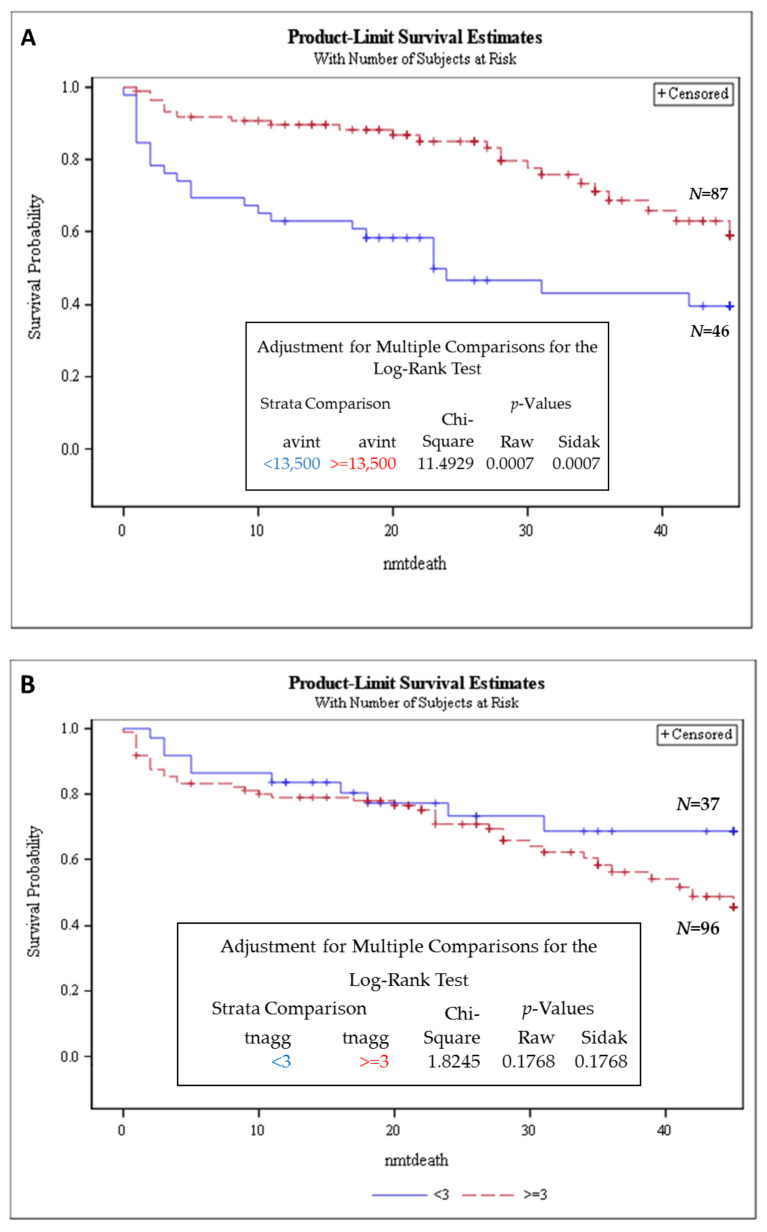
Average intensity (proportional to telomere length) and number of telomere aggregates is highly prognostic in MM group. Patients with telomere average intensity below 13,500 had significantly shorter OS when compared to patients above 13,500 (*p* = 0.007) (**A**). The same was observed for patients with number of telomere aggregates equal to or higher than 3 (**B**). Kaplan–Meier survival analysis, using the log-rank test, was performed only for the MM cohort. The telomere average intensity (<13,500 a.u) and number of telomere aggregates (≥3) (*p* = 0.17, not significant) threshold identified a subset of MM patients with inferior survival. dx—diagnosis; Strata—groups; raw *p*-values—*p*-values without adjustments; Sidak *p*-value—*p*-value for multiple comparisons. nmtdeath refers to months to dying censored. Censored refers to cases that were followed up to that point but did not die. Number of patients in each arm is indicated in the figure.

Next, using Cox regression analysis we investigated the prognostic value of the telomere parameters just in the MM group. The two independent predictors for overall survival were average intensity and number of telomere aggregates (*p* = 0.0003 and 0.02, respectively) (Supplementary Table S3). The cutoff threshold of 13,500 a.u for average intensity identified a subset of MM patients with inferior survival (Figure 4A). MM patients with a lower average intensity (below the threshold) had shorter OS as compared to those with a higher average intensity (*p* = 0.0007). Moreover, MM patients with a higher (↑) number of telomere aggregates (≥3) had shorter OS as compared to a lower number of telomere aggregates (Figure 4B). The telomere aggregate threshold ≥ 3, though not significant, points to a possible survival trend. Here, we report that telomere-related genomic instability correlates with disease aggressiveness and identifies high-risk subgroups in SMM and MM. In particular, average intensity and total intensity were associated with shorter overall survival for SMM and MM patients. Telomere length below 13,500 arbitrary units and an increased number of telomere aggregates (≥3) could be used as a prognostic factor to identify high-risk MM patients.

## 4. Discussion

Three-dimensional telomere analysis measures the level of genomic instability in single cells. Specific telomere profiles are associated with disease stability vs. progression [18,21,22,23,24,25]. Critically short telomeres, telomere dysfunction, and fusions have all been correlated with disease progression [18,25,27,28,29]. Therefore, telomere profiling may represent both a clinically useful prognostic tool and a potential guide for therapeutic intervention [23]. In current clinical practice, patients diagnosed with MGUS or SMM are monitored, but not treated. In this study, we evaluated bone marrow samples from a cohort of 214 patients diagnosed with MGUS (54), SMM (24), and MM (136) having a clinical follow-up of at least 60 months. We found several significant differences in the telomere parameters of the three disease stages, corroborating with previous observations performed by Yu et al. (2019) [18] and Klewes et al. (2013) [19]. In the SMM patient group, five different telomere parameters identified patients with stable or progressive disease and allowed us to stratify this group of SMM patients into high-risk SMM versus low-risk SMM. Similar results were observed for MM patients. This risk stratification has the potential to guide evidence-based treatment decisions of SMM patients with a high risk of progression or MM patients with active disease.

In Cox’s proportional hazards analysis, the average intensity and total intensity associated with shorter overall survival in MM (Figure 3 and Table 3), setting the average intensity threshold as 13.500 a.u., provided a clear differential OS of patients below and above the set threshold in the survival curve (Figure 3). A decrease in telomere length in MM compared to normal controls has been described in other studies. Wu et al. (2003) investigated telomere length in CD138^+^ flow-sorted cells from the bone marrow of 115 MM patients (newly diagnosed or relapsed) and seven healthy donors [30]. The results showed significantly reduced telomere length in MM patients compared to the telomere length in plasma cells from healthy donors [30]. Cottliar et al. (2003) studied bone marrow (BM) cells from 31 patients with MM and two with MGUS. They also observed a reduction in telomere length in MM patient samples at diagnosis and during relapse, but they noticed that telomere length (in BM cells) was restored after disease remission [31].

Interestingly, different telomere parameters differentiated MGUS from SMM or MM, but no common parameter emerged for the whole disease spectrum. This lack of a common parameter could be due to disease heterogeneity and clonal evolution at each stage. When Bolli et al. (2018) characterized the genomic landscape of high-risk SMM using whole-genome sequencing, they observed that cytogenetic, mutational, and rearrangement profiles were very similar to those described for MM [32]. The role of risk factors used in MM or best cutoffs in defining high-risk SMM remains under investigation. Our data show two distinct subpopulations inside of SMM and MM groups associated with a more aggressive disease, which is an important finding, since SMM patients currently are not usually treated until clinical symptoms of progression to MM appear. The subpopulation groups of each of these two stages of the disease were associated with different levels of genomic instability. This stratification has the potential to identify SMM and MM patients that will benefit from immediate treatment decisions [33,34].

We observed in the multivariate analysis that total and average intensity were associated with shorter overall survival in the SMM and MM groups. On the other hand, when we considered the aggressiveness in the MM group alone (stable vs. progressive disease), another parameter, namely number of telomere aggregates, emerged in place of the total intensity parameter (Supplementary Table S3). Figure 4B shows a clear separation in the survival curve beyond 60 months for a telomere aggregates threshold of 3 or higher.

To our knowledge, there is only one previous study evaluating the prognostic role of telomere length in myeloma [16]. Hyatt et al. (2017) [16] studied 61 patients with MGUS and 134 patients with MM using single telomere length analysis (STELA) and performed multivariate analysis on 113/131 (86.3%) MM samples. In their model, the analysis included the mean telomere length, gender, age, ISS sub-groups, and the telomere dysfunction threshold (3.81 kb). The most important prognostic factor in their study was the International Staging System (ISS) stage followed by age and telomere length below 3.81 kb. After adjustment for ISS and age, they also found that telomere length < 3.81 kb was associated with significantly shorter survival [16]. The present study is the first to provide a comprehensive spatial analysis of the 3D telomere parameters across MM stages and disease aggressiveness, and to identify subpopulations within the SMM and MM patient groups. Our study shows that lower average intensity (telomere length below 13,500 a.u) and an increased number of telomere aggregates decrease OS and could be used as a prognostic factor to identify high-risk myeloma patients.

Dynamic genomic instability is known to contribute to disease progression and patient response to therapy in MM [35,36]. Telomere molecular and structural characteristics have been shown to hold useful information regarding disease aggressiveness and may serve as a potential guide for therapeutic intervention [23,24,25,36,37,38]. Indeed, some current therapeutic approaches aim to create synthetic lethal interactions in MM cells [39,40]. Neri et al. (2011) showed that inhibition of the 26S proteasome impaired homologous recombination-mediated repair of DNA damage in MM cells, resulting in synthetic lethality when combined with PARP inhibitors [40]. Botrugno et al. (2019) also identified additional synthetic lethal approaches, beyond the PARP1, where the combination of DNA damaging agents, commonly used in the clinic to treat MM, could be associated with novel interventions to prevent cells from repairing DNA and hence trigger apoptosis [41]. Bajpai et al. (2016) revealed that MM cells that survive glutamine withdrawal enhance expression and binding of BIM to BCL-2. This binding can increase the response to BH3 mimetics, such as venetoclax [42]. On the other hand, it is known that telomerase activity is found in 90% of the newly diagnosed and relapsed MM patients. This seems contradictory, when we show a high number of short telomeres in MM patients both at diagnosis and during progression. However, Xu et al. (2003) showed that MM patients can have short telomeres as well as telomerase activity, supporting the concept of critically short telomeric DNA protection by telomerase [30]. In addition, the inhibition of telomerase activity by GRN163L, an oligonucleotide-targeting RNA component of telomerase, has been shown to be effective in treating MM both in vitro and in vivo, highlighting another approach that could be used to treat patients with telomere dysfunction [38].

## 5. Conclusions

In summary, the crucial determinant to improve the long-term outcomes for MM patients is the early identification of patients with the high-risk form of the disease. Here, we provide strong evidence for telomere signatures as a prognostic factor for disease aggressiveness at diagnosis. With better understanding of the ongoing genomic instability present in myeloma and its precursor lesions, newer targeted treatment strategies can be developed to improve the patient prognosis and, hopefully, better outcomes.

## Figures and Tables

**Table 1:** Basic clinical characteristics of the study population assessed at time of diagnosis.

Groups	MGUS	SMM	MM
Number of Patients	54	24	136
Basic Clinical Characteristics			
Age	66.6 ± 10.0	70.8 ± 10.0	67.5 ± 11.5
BMPC (%)	2.8 ± 1.3	18.1 ± 6.3	37.9 ± 28.2
M-protein	5.5 ± 4.5	17.1 ± 10.6	26.9 ± 21.4
IgG (%)	12.5	25.5	30.9
IgA (%)	3.6	7.7	9.9
IgM (%)	3.3	0.7	0.3
Lytic lesions (%)	0/54 (0%)	0/21 (0%)	86/135 (63.7%)
Cytogenetic information			
Patients with t(11;14) (%)	1/36 (2.7%)	N/A	20/25 (80.0%)
Patients with t(4;14) (%)	1/8 (12.5)	N/A	8/19 (42.1%)
Patients with del(14q1.3)/13qter (%)	0/8 (0%)	N/A	3/19 (15.8%)

BMPC indicates the degree of bone marrow plasma cell infiltration; M-protein indicates the serum level of myeloma protein. IgG, IgA, and IgM indicate the percentage of patients per cohort with each of the 3 isotypes of immunoglobulin heavy chain as the predominant isotype; n.d. is the percentage of patients whose immunoglobulin isotype data were unavailable. Numbers represent average values along with the maximal variance within them.

**Table 2:** Significance of telomere parameters in MGUS, SMM, and MM at diagnosis and after five-year follow-up.

Telomere Parameters	MGUS vs. MM (All Cases)	MGUS vs. SMM (All Cases)	SMM (All Cases) vs. MM (All Cases)	SMM Stable (stb) vs. SMM with High Risk to Progression (prg)	MM Stable (stb) vs. MM with Progressive Disease (prg)
Telomere numbers	*p* = 0.0193 (↑ MGUS × MM ↓)	ns	ns	*p* ≤ 0.0001 (↓ stb × prg ↑)	*p* ≤ 0.0001 (↓ stb × prg ↑)
Total telomere intensity	ns	*p* = 0.0370 (↓ MGUS × SMM ↑)	*p* = 0.0019 (↑ SMM × MM ↓)	*p* ≤ 0.0001 (↓ stb × prg ↑)	ns
Average telomere intensity	ns	*p* = 0.0009 (↓ MGUS × SMM ↑)	*p* = 0.0097 (↑ SMM × MM ↓)	*p* = 0.0493 (↓ stb × prg ↑)	*p* ≤ 0.0001 (↑ stb × prg ↓)
Telomere aggregates	ns	ns	ns	*p* = 0.0014 (↓ stb × prg ↑)	*p* = 0.0001 (↓ stb × prg ↑)
*a/c* ratio	*p* = 0.0112 (↓ MGUS × MM ↑)	ns	ns	*p* ≤ 0.0001 (↓ stb × prg ↑)	*p* ≤ 0.0001 (↓ stb × prg ↑)
Nuclear volume	ns	*p* = 0.05 (↓ MGUS × SMM ↑)	ns	*p* = 0.0033 (↓ stb × prg ↑)	*p* ≤ 0.0001 (↓ stb × prg ↑)
Telomeres per nuclear volumes	ns	ns	ns	ns	ns

Stb—stable; prg—progression; ns—not significant; up arrow (↑) refers to increase; down arrow (↓) refers to decrease. Indolent SMM patients remained stable for 5 years, while high-risk SMM (SMM progression) patients progressed to full stage multiple myeloma within 1 to 3 years from point of diagnosis. MM patients were classified as progressive if they died from the disease.

**Table 3:** Cox’s proportional hazards modelling of telomere signals adjusted by age and diagnosis.

Analysis of Maximum Likelihood Estimates								
Parameter	DF	Parameter Estimate	Standard Error	Chi-Square	Pr > ChiSq	Hazard Ratio	95% Hazard Ratio Confidence Limits	
AVINT	1	−0.45436	0.13470	11.3781	0.0007	0.635	0.488	0.827
TOTIN	1	0.04296	0.02112	4.1358	0.0420	1.044	1.002	1.088
MMDX	1	2.07806	0.60041	11.9789	0.0005	7.989	2.463	25.916
SMMDX	1	2.01007	0.75049	7.1736	0.0074	7.464	1.715	32.492
AGE	1	0.04876	0.01310	13.8605	0.0002	1.050	1.023	1.077

## Data Availability

The data presented in this study are available in this article (and supplementary material).

## Supplementary Materials

The following are available online at https://www.mdpi.com/article/10.3390/cancers13081969/s1, Figure S1: Immunostaining and Q-FISH in CD56^+^ and CD138^+^ malignant plasma cells (A–D). Table S1: Clinical data for participating patients. Table S2: Basic clinical characteristics of the study SMM and MM subpopulation. Table S3: Multivariate modelling of telomere signals by age and diagnosis using only the MM group.
