# Supplementary material for: Telomere Architecture Correlates with Aggressiveness in Multiple Myeloma
Source: Cancers (Basel). 2021 Apr 19;13(8):1969. doi: 10.3390/cancers13081969 (PMC8073772; doi:10.3390/cancers13081969)
Supplement: Supplementary file 1 [file cancers-13-01969-s001.zip › cancers-1156866-supplementary.pdf]

Supplementary Material: Telomere Architecture Correlates with Aggressiveness in Multiple Myeloma

Aline Rangel-Pozzo, Pak Lok Ivan Yu, Sadhana LaL, Yasmin Asbaghi, Luiza Sisdelli, Pille Tammur, Anu Tamm, Mari Punab, Ludger Klewes, Sherif Louis, Hans Knecht, Adebayo Olujohungbe and Sabine Mai

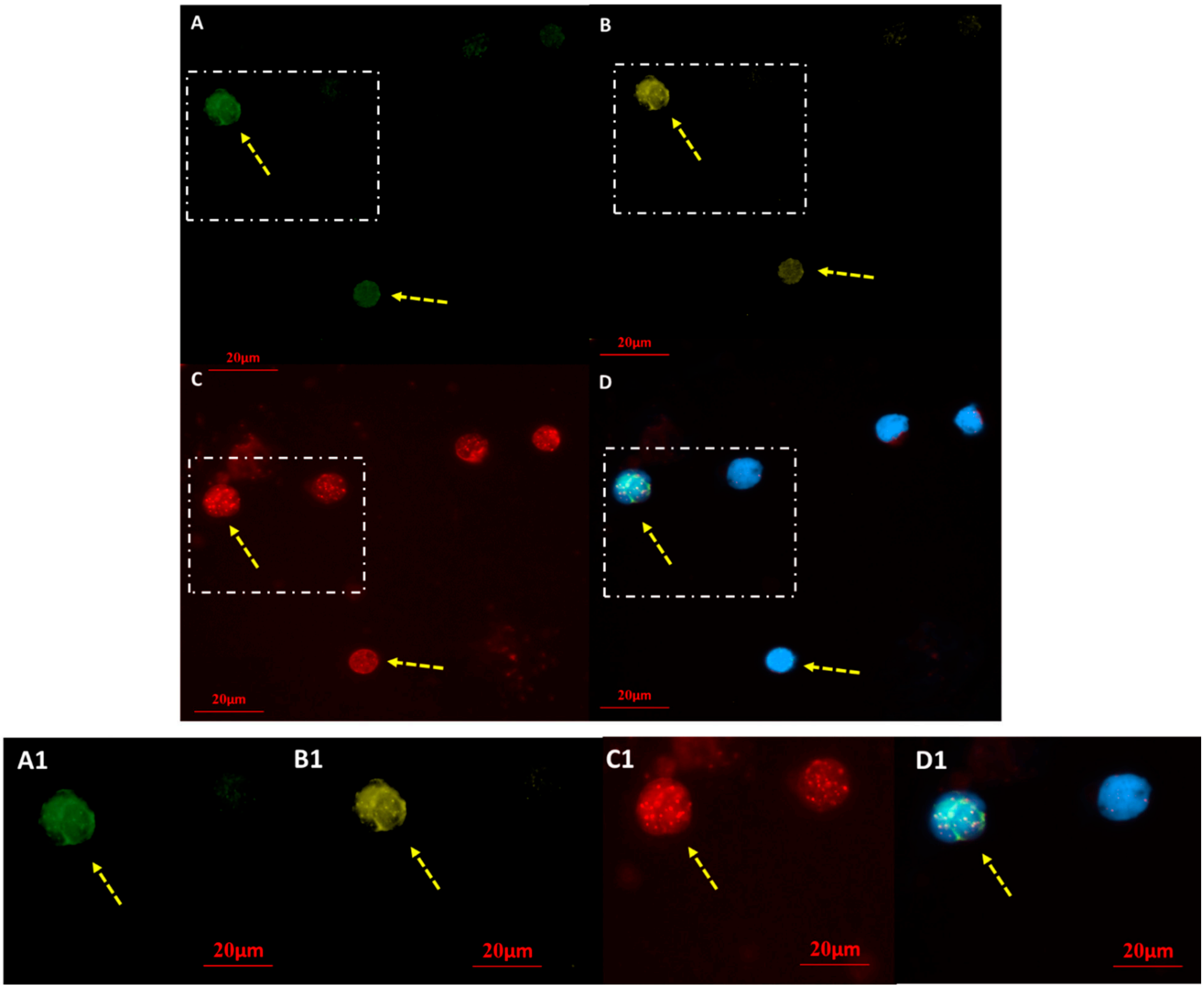

Figure S1. Immunostaining and Q-FISH in CD56+ and CD138+ malignant plasma cells (**A**–**D**). (**A**) CD56+ Myeloma cells fluoresce green with Alexa Fluor® 488 labelled anti-CD56 antibody, (see yellow arrows), and normal cells remain unstained. (**B**) CD138+ Myeloma cells stain yellow with Alexa Fluor® 594 labelled anti-CD138 antibody (see yellow arrows) while normal cells remained unstained. (**C**) The telomeres, hybridized with Cy3-labeled PNA probes, appear as red dots. (**D**) Merged image with Alexa Fluor® 488, Alexa Fluor® 594, Cy3-labeled PNA probes and the nuclei counterstained with DAPI (blue). Enlarged boxes are shown in (**A1**–**D1**). Their dual positive CD56 and CD138 staining can differentiate myeloma cells from normal lymphocytes and non-malignant plasma cells.

Table S1. Clinical data for participating patients.

Patient Sample	Diagnosis	Age	BMPCS %	IgG	IgA	IgM	Light Chain Isotype		Serum Free Light Chain			M Band	Lytic	t(11;14)	t(4;14)	del13q14/13qter
							Kappa	Lambda	Kappa	Lambda	Ratio	g/L	Lesions			
1	SMM	71	27	34.8	0.15	0.07	Yes		-	-	-	31	No	-	-	-
2	MM	80	3.2	8.61	6.32	1.18	IgA		69.8	22.6	3.09	3	No	-	-	-
3	MM	51	63	106	0.18	0.09	Yes		84.9	5.4	15.72	82	No	-	-	-
4	MM	58	32	27.8	0.38	0.05		Yes	12.2	610	0.02	20	No	-	-	-
5	MM	86	46	72.1	0.27	0.11	Yes		505	6.66	75.83	78	No	-	-	-
6	MM	81	37.6	45.5	0.3	0.2		Yes	0	-	0	34	No	-	-	-
7	MM	71	5.6	19.3	3.47	0.94	Yes		58.4	21.4	2.73	4	Yes	-	-	-
8	MM	74	85.4	61.2	0.28	0.18	Yes		526.5	6.29	83.7	45	Yes	-	-	-
9	MM	54	27.4	56.3	0.37	0.26	Yes		552.5	6.01	91.93	56	Yes	-	-	-
10	MM	75	16.6	33.8	1.95	0.3		Yes	13.5	677.5	0.02	25	Yes	-	-	-
11	MM	51	33	9.57	0.6	0.49	Yes		9.9	16.6	0.6	6	No	-	-	-
12	MM	81	36.6	3.05	20.8	0.04	Yes		737.5	11	67.5	19	No	-	-	-
13	MGUS	64	3	9.01	10.2	0.43	Yes		35.3	11.6	3.04	12	No	-	-	-
14	MM	93	25.4	14.8	0.24	0.08	Yes		17.8	4.95	3.6	14	Yes	-	-	-
15	MGUS	36	2.6	32	4.64	1.2		Yes	N/D	N/D	N/D	3	No	-	-	-
16	MM	86	10.4	2.03	10.9	0.7	Yes		7.79	10.5	0.74	12	No	-	-	-
17	MM	55	42.2	9.48	1.26	0.61		Yes	8.89	4050	0	7	Yes	-	-	-
18	MM	70	24.8	8.29	0.55	0.19	Yes		2215	12.3	180.08	7	Yes	-	-	-
19	MM	85	26.8	21.6	0.29	0.06	Yes		975	6.96	140.09	27	Yes	-	-	-
20	MM	61	62.6	4.26	74.3	0.24	Yes		1985	6.59	301.21	51	No	-	-	-
21	MM	75	35.2	13.1	0.26	0.11	IgG		-	-	120	10	No	-	-	-
22	MM	71	18.2	3.69	0.64	0.16		Yes	3.48	144	0.02	10	No	-	-	-
23	MM	69	12.4	4.04	29.6	0.27	Yes		30.1	9.21	3.27	28	Yes	-	-	-
24	MGUS	76	3.4	8.2	4.81	2.19		Yes	27	12.6	2.14	9	No	-	-	-
25	MM	82	49	4.33	0.12	0.22		IgG	24.4	132	0.19	2	Yes	-	-	-
26	MM	59	85.6	1.74	98.4	0.1		Yes	3	3700	0	52	Yes	-	-	-
27	MM	62	20.2	102	0.17	0.04	Yes		2012.5	6	3354	94	Yes	-	-	-
28	MGUS	64	4	8.61	5.91	0.98		Yes	6.31	37.2	0.17	9	No	-	-	-
29	MM	54	2.8	7.95	0.93	0.56	Yes		2455	6.2	395.97	12	Yes	-	-	-
30	MM	59	3	6.52	0.68	0.47	Yes		440	6.78	64.9	7	Yes	-	-	-
31	MM	56	29.8	48.6	0.32	0.45	Yes		2650	2.64	1003.79	36	Yes	-	-	-
32	SMM	57	6.2	13.8	1.86	2.47	Yes		22.4	8.02	2.79	7	No	-	-	-
33	MM	70	50.8	64.5	0.15	0.11		Yes	-	-	130	48	No	-	-	-
34	MM	57	48.6	30	0.92	0.73		Yes	11.3	542.5	0.02	24	Yes	-	-	-
35	MM	75	66	25	45.7	0.48	0.65		-	-	140	8	Yes	-	-	-
36	MM	59	91.6	0.95	37.9	0.06	Yes		-	-	166	37	Yes	-	-	-
37	MM	55	0.8	25.4	0.71	0.18	Yes		620	6.4	96.88	19	Yes	-	-	-
38	MM	58	0.2	5.38	0.61	0.4			<3	6.18	<0.49	9	No	-	-	-

39	MM	74	36.2	0.21	29.27	0.47	Yes		>4500	9.61	>468.26	26	Yes	-	-	-
40	MM	63	2.8	N/A	N/A	N/A	Yes		31.9	12.4	2.57	10	Yes	-	-	-
41	MM	68	48.6	41.5	0.39	0.17	Yes		102	7.55	13.51	33	Yes	-	-	-
42	MM	64	6.6	32	1.34	1.17		Yes	12	80.1	0.15	22	No	-	-	-
43	MM	61	45.6	66.3	0.22	0.27	Yes		727.5	5.66	128.53	53	Yes	-	-	-
44	MM	70	16.2	22.8	0.86	0.69	Yes		622.5	10.9	57.11	15	No	-	-	-
45	MM	81	14.6	6.22	11.6	0.89	Yes		41.6	14.1	2.95	12	Yes	-	-	-
46	MM	64	33	26.2	0.54	0.28	Yes		1302.5	6.78	192.11	19	No	-	-	-
47	MM	75	61.6	4.98	0.4	0.34	Yes		>4500	12.1	>371.9	12	Yes	-	-	-
48	MGUS	58	1.8	16	1.51	0.69	Yes		<3.3	6.5	0.51	2	No	-	-	-
49	MM	51	7.4	11.9	8.05	0.38	Yes		15.8	10.6	1.49	2	No	-	-	-
50	MGUS	85	1.2	14.7	2.06	0.61	Yes		69.4	29.5	2.35	2	No	-	-	-
51	MM	60	61.8	1.75	77.2	0.04		Yes	4.19	>4050	0	61	Yes	-	-	-
52	MM	70	15.6	4.09	0.37	0.29		Yes	<3.3	555	0.01	7	Yes	-	-	-
53	MM	52	1.3	14.6	2.26	1.1	Yes		14.1	12.3	1.15	3	No	-	-	-
54	MM	64	69	4.77	0.38	0.11		Yes	12.6	15.9	0.79	2	Yes	-	-	-
55	MM	71	**	34.5	0.24	0.36		Yes	18.1	465	0.04	26	No	-	-	-
56	MM	45	93	38.9	0.93	0.65	Yes		-	-	-	29	No	-	-	-
57	MM	65	45	5.51	22.6	0.12	Yes		>4500	94.3	>47.72	13	Yes	-	-	-
58	MM	81	91.2	73.9	<0.07	0.05	Yes		-	-	<100	72	No	-	-	-
59	MGUS	54	1.8	11.3	2.17	1.27	Yes		15.8	14.1	1.12	2	No	-	-	-
60	MM	75	9.8	40.3	0.2	0.4	Yes		12.5	6.54	1.91	31	No	-	-	-
61	MM	77	34	75.1	0.16	0.1	Yes		0.01	-	<100	63	Yes	-	-	-
62	MGUS	39	3	11	9	10	0.82		-	-	-	2	No	-	-	-
63	MM	73	9.2	6.3	14.6	0.33	Yes		980	25.1	39.04	17	Yes	-	-	-
64	MM	62	3	12.2	3.84	0.73		Yes	11.5	462.5	0.03	12	Yes	-	-	-
65	MM	83	12.8	12.3	3.82	0.6			11.4	200	<100	8	Yes	-	-	-
66	MGUS	84	5.4	8.26	4.05	14.1	Yes		>4500	402.5	>11.18	9	No	-	-	-
67	MM	70	88.8	4.47	33.3	0.3	Yes		117	8.82	13.27	20	Yes	-	-	-
68	SMM	72	54.8	47.3	0.47	0.24		Yes	10.4	13.8	0.75	38	No	-	-	-
69	MM	56	26.6	25.7	0.17	0.18	Yes		23.4	8.87	2.64	19	Yes	-	-	-
70	MGUS	60	5.6	9.31	5.83	0.96	Yes		37.1	14.1	2.63	1	No	-	-	-
71	MM	37	73	7.82	1.14	0.36	Yes		7.58	9	0.84	3	Yes	-	-	-
72	MM	63	15.4	8.01	0.6	0.48	Yes		94.3	7.34	12.85	12	Yes	-	-	-
73	MGUS	73	1.6	9.16	1.67	7.13	Yes		29.7	16.3	1.82	2.1	No	-	-	-
74	MM	74	32.6	76.2	<0.07	<0.04		Yes	<3	837.5	0	58	No	-	-	-
75	MM	81	14.6	16.1	17.9	0.51		Yes	41.7	104	0.4	9	No	-	-	-
76	MM	63	2.4	6.48	1.62	1.12	Yes		7.98	7.42	1.08	8	No	-	-	-
77	SMM	82	9.2	29.9	0.46	0.4	Yes		17.8	11.8	1.51	17	No	-	-	-
78	MM	60	44.6	5.53	0.42	0.54		Yes	6.22	377.5	0.02	2	Yes	-	-	-
79	MM	80	88	4.59	45.9	0.09	IgG and IgA		29.4	6.79	4.33	39	-	-	-	-

80	MM	67	23	10.6	1.1	0.4	Yes		-	-	<100	4	No	-	-	-
81	MM	62	46	34.3	0.67	0.18		Yes	<2.7	108	<0.03	29	Yes	-	-	-
82	MM	54	40.8	53.4	<0.07	0.15	Yes		11	4.61	2.39	48	Yes	-	-	-
83	MM	50	4	12.7	4.98	0.89	Yes		86	54.4	1.58	7	No	-	-	-
84	MM	75	42.8	45.8	0.4	0.33		Yes	13.1	87.1	0.15	43	No	-	-	-
85	MM	48	14.8	57.4	2.48	0.26		Yes	20.4	2275	0.01	57	Yes	-	-	-
86	MM	76	6.8	45.5	0.22	0.26		Yes	6.48	622.5	0.01	33	Yes	-	-	-
87	SMM	74	11.4	12.7	3.92	0.89	Yes		21.5	40	0.54	7	No	-	-	-
88	MM	77	89.6	45.8	0.33	0.4		Yes	9.8	12	0.82	39	No	-	-	-
89	MM	50	18.6	32.1	3.31	1.37	Yes		79	17.6	4.49	23	Yes	-	-	-
90	MM	70	13.2	10.5	1.46	0.55		Yes	9.98	270	0.04	10	Yes	-	-	-
91	MM	67	34.4	6.95	37.8	0.14	Yes		12.4	<2.6	>4.77	24	No	-	-	-
92	MM	52	66.6	5.58	0.76	0.18		Yes	9.94	>4400	<0.00	6	Yes	-	-	-
93	MM	68	80.4	64.1	0.15	0.14	Yes		1137.5	2.6	437.5	55	No	-	-	-
94	MM	68	55.8	41.5	0.18	0.07	Yes		45.6	3.46	13.18	29	Yes	-	-	-
95	MM	64	23.4	56.3	0.34	0.24	Yes		42.5	3.82	11.13	49	Yes	-	-	-
96	MM	68	85	66.9	0.95	0.34	Yes		680	2.6	261.54	56	Yes	-	-	-
97	SMM	66	16.2	36.9	0.47	0.37		Yes	10.2	800	0.01	31	No	-	-	-
98	MGUS	69	2.8	5.73	17.1	2.06	Yes		11.5	5.42	2.12	17	No	-	-	-
99	SMM	55	8.2	29.5	0.25	0.46		Yes	<2.7	11.7	0.23	25	No	-	-	-
100	MM	78	30.2	74.6	0.13	0.11	Yes		1620	2.6	623.08	62	Yes	-	-	-
101	MM	68	12.8	40.5	0.21	0.09	Yes		-	-	<100	32	Yes	-	-	-
102	MM	81	5.8	42.2	0.2	0.15	Yes		970	17.2	56.4	41	Yes	-	-	-
103	MM	81	65.2	31.3	0.08	0.21		Yes	<2.7	972.5	<100	35	Yes	-	-	-
104	MGUS	51	5.2	8.8	0.97	5.84		Yes	2.83	12	0.24	4	No	-	-	-
105	MM	67	52	85.8	0.07	0.11		Yes	12.5	277.5	0.05	84	No	-	-	-
106	MM	75	13.6	32.01	0.55	0.09		Yes	<2.7	672.5	<100	24	Yes	-	-	-
107	MM	70	34.8	10	0.6	0.22	Yes		2.7	7.8	0.35	50	Yes	-	-	-
108	MM	45	68.6	2.21	72.6	0.31		Yes	<2.7	>4400	<100	58	No	-	-	-
109	MM	70	12.6	7.65	11.2	1.13		Yes	37.9	327.5	0.12	12	No	-	-	-
110	MM	74	43.6	55.7	0.09	0.11	Yes		145	3.5	41.43	43	No	-	-	-
111	SMM	88	12	6.05	24.5	0.22	Yes		143	16.9	8.46	18	No	-	-	-
112	SMM	79	15	24.8	2.1	1.06	Yes		272.5	16.3	16.72	16	-	-	-	-
113	MM	58	10.8	8.32	0.62	0.26	Yes		3.28	5.09	0.64	3	Yes	-	-	-
114	SMM	79	9.8	19.7	1.04	0.68		Yes	13.5	33.6	0.4	12	No	-	-	-
115	MM	68	15	10.6	0.5	0.28			12.8	5.58	2.29	12	No	-	-	-
116	SMM	69	12	15.4	2.9	0.47		Yes	8.94	10.3	0.87	3	No	-	-	-
117	SMM	82	8.4	30	0.11	0.06	Yes		14.3	6.08	2.35	20	No	-	-	-
118	MM	63	94	2.09	0.09	0.06		Yes	<3.15	>4200	<100	10	Yes	-	-	-
119	SMM	63	18.2	2.96	14	0.8		Yes	3.73	213	0.02	11	No	-	-	-
120	SMM	87	20	42.5	0.24	0.16	Yes		325	8.85	36.72	28	No	-	-	-
121	MGUS	67	5	6.64	12.7	0.21		IgA	<3.15	70.9	0.04	5	No	-	-	-

122	SMM	83	9.6	34.5	0.91	0.8	Yes		24.3	2.45	9.92	23	No	-	-	-
123	MM	62	14	29.8	0.28	0.52	Yes		762.5	2.61	292.15	17	Yes	-	-	-
124	MM	58	20.6	18.3	0.44	0.14		IgG	7.42	11	0.08	10	Yes	-	-	-
125	MGUS	57	2	11.1	1.53	2.67	IgM		8.51	7..92	1.08	1	No	-	-	-
126	MM	66	35	12.2	0.99	1.67	IgG		27.5	22.3	1.23	3	Yes	-	-	-
127	MGUS	65	9.8	3.99	0.07	13.5	IgM		>4725	325	>14.54	5	No	-	-	-
128	MM	47	30	65.3	0.8	0.39	Yes		32.4	12.5	2.59	53	No	-	-	-
129	SMM	61	11.4	27	2.53	0.27	IgG		29.3	21.1	1.39	15	No	-	-	-
130	MM	81	40	3.05	0.4	0.09			>4950	3.45	>1434.78	12	No	-	-	-
131	SMM	86	13	30.5	0.47	0.42	IgG		43.5	8.52	5.11	25	N/A	-	-	-
132	MGUS	72	3	8.56	0.34	57	IgM	IgG	26.9	12.9	2.09	2	No	-	-	-
133	MGUS	73	4	14.1	3.35	0.73	IgG		-	-	-	6	No	-	-	-
134	SMM	80	23	27.2	1.19	1.25	IgG		419.22	9.08	46.17	14	No	-	-	-
135	SMM	62	20.2	46.5	0.15	0.39	IgG		17.2	5.65	3.04	38	No	-	-	-
136	MM	62	40	44.6	0.25	0.15	IgG		463.57	0.5	927.14	32	No	-	-	-
137	MM	62	13	62	42.7	<0.15	0.1		-	-	<100	12	Yes	-	-	-
138	SMM	75	15	16.2	26.9	3.47	0.37		-	-	-	4	No	-	-	-
139	MM	65	33	5.43	0.25	0.17	IgG		10.7	6.65	1.61	1	No	-	-	-
140	SMM	64	32	22	0.99	0.41		IgG	62.37	4.63	13.47	11	-	-	-	-
141	MM	83	17	2.84	0.14	0.07			1778	0.53	3354.7	Not present	Yes	-	-	-
142	MM	70	15	65	42.9	<0.15	0.1		-	-	<100	12	Yes	-	-	-
143	MM	58	22	58	42.7	<0.15	0.1		-	-	<100	15	Yes	-	-	-
144	SMM	58	20	26.2	36.6	0.77	0.37		-	-	-	5	No	-	-	-
145	MM	58	25	60.1	48.7	<0.15	0.1		-	-	<100	20	No	-	-	-
146	MM	59	12	60.9	42.9	<0.15	0.1		-	-	<100	11	No	-	-	-
147	MM	58	17	52	10	<0.15	0.1		-	-	<100	14	No	-	-	-
148	SMM	45	15	16.2	40	0.47	0.37		-	-	-	5	No	-	-	-
149	MGUS	59	5	10.5	9.6	10.5	0.98		-	-	-	2	No	-	-	-
150	MM	90	20	60	42.7	<0.15	0.1		-	-	<100	14	Yes	-	-	-
151	MM	52	22	56	23	<0.15	0.1		-	-	<100	18	Yes	-	-	-
152	MM	55	27	63	34	<0.15	0.1		-	-	<100	20	Yes	-	-	-
153	SMM	56	14	20	23	0.47	0.37		-	-	-	7	No	-	-	-
154	MM	71	30	60	42.7	<0.15	0.1		-	-	<100	22	Yes	-	-	-
155	MM	62	33	66	22.7	<0.15	0.1		-	-	<100	21	Yes	-	-	-
156	MGUS	82	2	5.7	0.57	6.58			35.08	11.81	2.97	3.4	No	No	-	-
157	MGUS	52	2	6.7	5.13	0.61		Yes	10.54	203.69	0.05	2.1	No	No	-	-
158	MGUS	75	2.5	12.8	2.34	1.59	Yes		31.06	21.06	1.47	4.3	No	No	-	-
159	MGUS	50	2	8.9	1.96	6.99	Yes		2.31	1.19	1.94	6	No	No	-	-
160	MGUS	72	2	19.3	0.73	0.38		Yes	13.51	15.32	0.88	9.4	No	No	-	-
161	MGUS	84	3	16.9	1.71	0.96	Yes		21.33	21.82	0.97	7.4	No	No	-	-
162	MGUS	64	2	16.3	2.26	0.89	Yes		25.47	15.93	1.6	4.8	No	No	-	-

163	MGUS	53	3	14.9	2.16	1.71		Yes	10.49	12.51	0.84	5.4	No	No	-	-
164	MGUS	56	3	13.6	0.49	0.39		Yes	0.08	7.56	91.37	7.9	No	No	No	No
165	MGUS	69	4	10.4	9.53	1.39	Yes		28.3	24.13	1.17	5	No	No	-	-
166	MGUS	73	2	17	1.34	1.55	Yes		44.04	12.06	3.65	8.6	No	No	-	-
167	MGUS	74	0	15.1	1.5	4			-	-	-	7	No	No	-	-
168	MGUS	71	2	27.1	1.23	1.08	Yes		6.39	1.58	4.04	16	No	No	-	-
169	MGUS	73	1.2	14.6	2.18	1.15			18.13	13.32	1.36	6.2	No	No	-	-
170	MGUS	69	4	20	3.5	0.75	Yes		3.65	3.72	0.98	13.6	No	No	-	-
171	MGUS	64	1.5	11.3	1.49	0.63			12.55	13.58	0.92	5.2	No	No	-	-
172	MGUS	69	2	15	2.24	0.53		Yes	26.24	45.53	0.58	4.7	No	No	-	-
173	MGUS	67	3	15.7	0.23	0.24	Yes		10.58	6.27	1.69	13.4	No	No	-	-
174	MGUS	75	2	22.6	0.84	0.56			13.58	9.87	1.38	15.8	No	Yes	No	No
175	MGUS	74	2	15.3	1.69	0.31			22.02	33.92	0.65	7	No	No	-	-
176	MGUS	63	3	13.7	2	1.05		Yes	18.5	26.68	0.69	7.5	No	No	-	-
177	MGUS	68	4	9	2.8	1.3		Yes	11.13	11.58	0.96	3.5	No	No	No	No
178	MGUS	83	2	16.8	1.38	0.71		Yes	17.16	21.47	0.8	13.7	No	No	No	No
179	MGUS	62	1.2	8.8	6	1.14		Yes	18.68	106.37	0.18	1.8	No	No	-	-
180	MGUS	42	1.5	8.3	1.37	1.13	Yes		11.84	116.89	0.1	3.4	No	No	-	-
181	MGUS	68	5	18	3.78	2.4	Yes		22.37	17.86	1.25	9.6	No	No	-	-
182	MGUS	72	4	11.3	2.18	1.37	Yes		23.74	19.94	1.19	3.5	No	No	No	No
183	MGUS	54	1	8.5	4.74	0.96		Yes	10.6	32.37	0.33	4.1	No	No	-	-
184	MGUS	83	1	9.3	10.3	0.82		Yes	22.48	27.08	0.83	4.6	No	No	-	-
185	MGUS	80	1	9.3	10.73	0.93		Yes	-	-	-	7.8±2.3	No	No	-	-
186	MGUS	69	5	11.6	3.36	1.46	Yes		22.17	13.83	1.6	0	-	-	-	-
187	MGUS	77	3	10.8	3.1	0.85	Yes		35.67	21.95	1.63	2.7	No	No	-	-
188	MGUS	64	5	13.9	2.64	0.88	Yes		22.31	14.8	1.5	8.6	No	No	Yes	No
189	MGUS	57	3-4	11.8	3.15	1.32		Yes	17.48	11.8	1.32	2.2	No	No	No	No
190	MGUS	61	3	10.8	0.82	0.78			-	-	-	6.4	No	No	-	-
191	MM	61	5	13.6	2.08	1		Yes	24.56	21.63	1.14	6.8	No	No	No	No
192	MM	60	2	11.6	2.22	1.43	Yes		47.02	23.68	1.99	5	No	No	-	-
193	MM	71	90	3.7	33.4	<0.05		Yes	10.96	2317.75	0	25.5	Yes	Yes	-	-
194	MM	37	90	76	<0.15	<0.05		Yes	10.46	3092.7	0	48	Yes	Yes	-	-
195	MM	85	90	3	0.25	<0.05		Yes	18.18	7909	0	1.8	Yes	Yes	No	No
196	MM	89	75	11.1	0.24	0.06	Yes		-	-	-	5.2	Yes	Yes	-	-
197	MM	75	95	5.9	0.34	<0.05		Yes	3.68	2295	0	3.4	No	No	No	No
198	MM	76	95-100	40.2	0.28	<0.05		Yes	13.78	5469	0	34.6	Yes	Yes	No	No
199	MM	71	95	61.6	<0.3	<0.05	Yes		710.34	16.01	44.37	60.3	Yes	Yes	-	-
200	MM	52	15	84.8	0.36	0.17	Yes		1529.4	13.45	113.7	42.6	Yes	Yes	No	No
201	MM	64	60	42.7	<0.15	0.1	Yes		297.78	12.24	24.33	35.1	Yes	Yes	Yes	No
202	MM	87	50	37.5	0.49	0.65			15.28	6.82	2.24	31	No	No	-	-
203	MM	74	25	24	0.4	0.31	Yes		757.99	3.53	214.73	19	Yes	Yes	Yes	No
204	MM	70	90	66.2	0.54	0.34	Yes		288.92	5.7	50.69	74.3	Yes	Yes	No	No

205	MM	60	70	3.1	43.6	0.16		Yes	12.64	0.02	821.51	36.9	No	No	Yes	No
206	MM	87	50	41.5	<0.15	0.13	Yes		691.17	3.09	223.68	29	Yes	Yes	Yes	Yes
207	MM	58	30-40	25.2	<0.15	0.12	Yes		32.52	3.82	8.51	21.9	No	No	Yes	No
208	MM	68	60	4.8	0.6	0.42	Yes		27300	17.38	1570.77	17	Yes	Yes	No	No
209	MM	89	15	24.1	0.17	0.16	Yes		57.45	7.99	7.19	26.2	Yes	Yes	-	-
210	MM	84	25	45.4	0.42	0.65	Yes		47.07	2.59	18.17	41	Yes	Yes	No	No
211	MM	69	90	59.2	0.25	0.16	Yes		4470.74	7.49	596.89	79	Yes	Yes	Yes	Yes
212	MM	60	80	60.6	<0.15	0.37	Yes		1292.13	8.8	146.83	67.3	Yes	Yes	Yes	No
213	MM	68	85	45.9	<0.15	<0.15		Yes	11.7	3361.35	0	44.7	Yes	Yes	No	No
214	MM	88	20	7.5	0.69	0.38			95.62	10.36	9.23	0	Yes	Yes	No	No

Table S2. Basic clinical characteristics of the study SMM and MM subpopulation.

Groups	SMM		MM	
	**Stable**	**High-risk**	**Stable**	**Progression**
Number of Patients	15	5	85	48
Basic Clinical Characteristics				
Age	69.9 ± 9.5	74.2 ± 11.8	65.2 ± 11.7	71.5 ± 9.8
BMPC (%)	3.4 ± 1.7	21.88 ± 18.8	36.2 ± 27.6	41.5 ± 30.0
M-protein	15.9 ± 7.1	19.4 ± 13.5	25.2 ± 21.5	30.6 ± 21.5
IgG (%)	23.5	30.48	30.5	29.6
IgA (%)	5.3	5.6	8.8	10.2
IgM (%)	0.8	0.4	0.3	0.3
Lytic lesions (%)	0	0	51/85 (60.0%)	88/134 (65.7%)
Cytogenetic information				
Patients with t(11;14) (%)	N/A	N/A	7/10 (70.0%)	11/14 (78.5%)
Patients with t(4;14) (%)	N/A	N/A	4/8 (50.0%)	3/9 (33.3%)
Patients with del(14q1.3)/13qter (%)	N/A	N/A	2/8 (25.0%)	0/9 (0%)

BMPC indicates the degree of bone marrow plasma cell infiltration; M-protein indicates the serum level of myeloma protein. IgG, IgA, and IgM indicate the percentage of patients per cohort with each of the 3 isotypes of immunoglobulin heavy chain as the predominant isotype; n.d. is the percentage of patients whose immunoglobulin isotype data were unavailable. Numbers represent average values along with the maximal variance within them.

Table S3. Multivariate modelling of telomere signals by age and diagnosis using only the MM group.

Analysis of Maximum Likelihood Estimates								
**Parameter**	**DF**	**Parameter Estimate**	**Standard Error**	**Chi-Square**	**Pr > ChiSq**	**Hazard Ratio**	**95% Hazard Ratio Confidence Limits**	
avint	1	-0.56609	0.15467	13.3946	0.0003	0.568	0.419	0.769
tnagg	1	0.30461	0.13345	5.2101	0.0225	1.356	1.044	1.761
Age	1	0.05144	0.01495	11.8432	0.0006	1.053	1.022	1.084

The measure of effect is the hazard ratio, which is the risk of failure (i.e., the risk or probability of suffering the event in question). If the hazard ratio is less than 1, then the predictor is protective (i.e., associated with improved survival). On the other hand, if the hazard ratio is greater than 1, then the predictor is associated with increased risk (or decreased survival). The *p* value shows statistically significant associations between the first column parameters with mortality. DF–degree of freedom, each predictor occupies 1 degree of freedom in the model. DF–degree of freedom, each predictor occupies 1 degree of freedom in the model. Avint–average intensity of telomere signals, tnagg–total number of telomere aggregates.
